# Is Bleeding Gastrointestinal Stromal Tumor Associated With Higher Mortality and Morbidity?

**DOI:** 10.7759/cureus.47398

**Published:** 2023-10-20

**Authors:** Rema Alrashed, Hussam AlHarbi, Bandar Ali, Alanoud Mubarah, Faisal AlGhamdi

**Affiliations:** 1 Department of Surgery, Prince Sultan Military Medical City, Riyadh, SAU; 2 Department of Surgery, AlKharj Armed Forces Hospital, Riyadh, SAU

**Keywords:** gastrointestinal bleeding, non-bleeding gist, bleeding gist, gist, gastrointestinal stromal tumors

## Abstract

Introduction

Gastrointestinal stromal tumor (GIST) is the most common primary mesenchymal tumor of the gastrointestinal tract. The clinical presentation of GIST varies widely and ranges from being asymptomatic to being a life-threatening emergency in the form of gastrointestinal bleeding or bowel obstruction. Multiple prognostic factors have been identified for GIST, including, most importantly, larger tumor size (>5 cm), higher mitotic activity (>5 per 50 high-power fields), rupture of the mass, site of the mass, and personal history of GIST.

Objective

In this paper, we aim to study bleeding in gastrointestinal tumors as a predictor for morbidity and mortality and investigate possible factors influencing bleeding in GIST.

Method

A retrospective study design was used. Electronic medical records of 39 patients diagnosed with GIST in Prince Sultan Military Medical City in Riyadh between January 2015 and December 2020 were retrieved. Normal variables were presented as mean and standard deviation, while non-normal variables were presented as median and interquartile range. Student t-test or Mann-Whitney test was used to compare quantitative data when appropriate. The distribution of recurrence and survival was plotted with the Kaplan-Meier curve and compared between the two groups (bleeding and non-bleeding groups) with the log-rank test. Factors affecting recurrence and mortality were assessed using univariable Cox regression.

Result

A total of 39 cases of GIST tumors were included in this study. Patients were categorized according to the presentation into two groups: patients with no bleeding (n= 28) and those presented with bleeding (n= 11). The gender distribution was equal. The mean age was 58.14± 12.46 in patients with non-bleeding GIST and 58.18± 16.01 in patients with bleeding GIST. The most common location of GIST was the stomach (22 cases, 56%). Neither group had any significant differences regarding age (P-value = 0.993), gender (P-value = 0.648), tumor location (P-value = 0.057), size (P-value = 0.250), cluster of differentiation (CD) markers, histological types (P-value = 0.692), and shape (P-value = 0.079). There was no significant difference in recurrence between both groups (log-rank P = 0.972). There was no significant difference in survival between both groups (Log-rank P = 0.506).

Conclusion

We concluded that bleeding GIST was not a significant predictor for recurrence rate or higher mortality. This can help in the debate of whether bleeding GIST should be added as an independent factor in risk stratification. Despite that, further studies are needed to identify more variables that can add more accuracy to the pre-existing risk stratification systems.

## Introduction

Gastrointestinal stromal tumor (GIST) is the most common primary mesenchymal tumor of the gastrointestinal tract. GIST arises from the interstitial cells of Cajal, which are considered the pacemakers of the intestinal wall's musculature [1]. It is believed that GIST results from a mutated KIT gene, which is a proto-oncogene encoding the receptor tyrosine kinase protein known as tyrosine-protein kinase KIT, cluster of differentiation 117 (CD117), or mast/stem cell growth factor receptor (SCFR), and/or platelet-derived growth factor receptor alpha (PDGFRA) polypeptide, leading to higher rates of cell division and eventually tumor [2]. GIST can develop anywhere along the gastrointestinal tract but is found most commonly in the stomach, small bowel, and colon, and rarely in the omentum, mesentery, and behind the peritoneum (retroperitoneal origin) [3]. The clinical presentation of GIST varies widely and ranges from being an incidental finding on the radiological or endoscopic work-up to being an emergency in the form of gastrointestinal bleeding or bowel obstruction. Gastrointestinal bleeding is a life-threatening clinical presentation of GIST. To establish a diagnosis of GIST, several imaging modalities can be used, including computed tomography (CT) scan, magnetic resonance imaging (MRI), and positron emission tomography (PET), but CT scan remains the preferred modality as it is the most sensitive tool for visualizing local and distant metastasis. Under histological examination, a positive CD117 marker is diagnostic in more than 94% of cases. The mainstay of treatment is surgical resection, but the use of imatinib, which is a small molecule inhibitor targeting multiple tyrosine kinases, including c-KIT, might be considered in patients with poor prognostic factors and those with metastasis as it improves survival and decreases recurrence when used as neoadjuvant and/or adjuvant therapy. Multiple prognostic factors have been identified for GIST, including most importantly larger tumor size (>5 cm), higher mitotic activity (>5 per 50 high-power fields), rupture of the mass, site of the mass, and personal history of GIST [4, 5]. Tumor rupture can result not only in direct spread of the tumor to the abdomen but also in bleeding, which increases the overall morbidity and mortality. In this paper, we aim to study bleeding in gastrointestinal tumors as a predictor for morbidity and mortality and investigate possible factors influencing bleeding in GIST.

## Materials and methods

Study design and settings

This study is a retrospective descriptive study that included a total of 39 adult patients diagnosed with GIST at Prince Sultan Military Medical City between January 2015 and December 2020.

The inclusion criteria were being older than 18 years of age, having provided consent to participate in the study, being residents of the Riyadh region, and having received a GIST diagnosis with conclusive histopathological findings.

The exclusion criteria were being younger than 18 years of age, not providing consent to participate in the study, residing outside the Riyadh region, and receiving a GIST diagnosis with inconclusive histopathological findings.

Data collection

Electronic medical records of patients diagnosed with GIST at Prince Sultan Military Medical City in Riyadh were retrieved for the period between January 2015 and December 2020. The variables included were patients' age, gender, clinical presentation (bleeding and non-bleeding GIST), radiological findings, histopathological findings with immunohistochemical markers such as CD117, DOG-1, DOG-7, CD34, SMA, desmin, and S-100 proteins, as well as surgical procedures. In November 2022, patients were followed up for recurrence or mortality. All patients of various nationalities who received a GIST diagnosis through imaging techniques, endoscopy, or excisional biopsy were included. Meanwhile, patients with inconclusive histopathological examinations were excluded.

Data management and statistical analysis

Statistical analysis was conducted using Stata 17 (Stata Corp, College Station, Texas). Quantitative data were assessed for normal distribution, and normal variables were reported as mean and standard deviation, while non-normal variables were presented as median and interquartile range. To compare quantitative data, the Student t-test or Mann-Whitney test was employed as appropriate. The distribution of recurrence and survival was illustrated using Kaplan-Meier curves and compared between the two groups (bleeding and non-bleeding) with the log-rank test. Factors influencing recurrence and mortality were examined through univariable Cox regression analysis. A P-value less than 0.05 was considered statistically significant.

Ethical consideration

All participants were volunteers. All data were kept confidential and used only for research purposes. Privacy was ensured for all of the included patients. The ethical approval was obtained from the ethical committee of Prince Sultan Military Medical City, Riyadh, Saudi Arabia.

## Results

A total of 39 cases of GIST tumors were included in this study, with an almost equal gender distribution. The age at diagnosis was between 40 and 60 years for 59% of the patients. The most common location for GIST was the stomach (22 cases, 56%), followed by the small bowel (8 cases, 20.5%). Other cases were found in the duodenum, rectum, retroperitoneum, liver, lower esophagus, and intra-abdominal locations with an unknown origin. The most prevalent manifestations included abdominal pain (46.2%), followed by upper or lower gastrointestinal (GI) bleeding (28.2%). In 5 patients, GIST was incidentally discovered during radiological or endoscopic investigations or intra-operatively. Additionally, 2 cases were identified histologically post-sleeve gastrectomy. An intraoperative case was found in the jejunum during pancreatic head resection as part of the Whipple procedure. The largest tumor diameter was observed in the esophagus, measuring 18 cm. Demographic and clinical data are detailed in Table 1. The clinical presentation of patients varied, including bleeding (n= 11), hematuria (n= 1), upper gastrointestinal bleeding (n= 6), and lower gastrointestinal bleeding (n= 4). Patients were categorized based on their presentation into two groups: those with no bleeding (n= 28) and those who presented with bleeding (n= 11).

**Table 1:** Demographics and clinical presentation of GIST patients (n = 39)

Demographic/clinical presentation		N (%)
Age	<40 years	2 (5.1%)
	40–49 years	13 (33.3%)
	50-60 years	10 (25.6%)
	>60 years	14 (35.9%)
Gender	Male	19 (48.7%)
	Female	20 (52.4%)
Tumor location	Duodenum	2 (5.1%)
	Stomach	22 (56%)
	Small bowel	8 (20.5%)
	Liver	1 (2.6%)
	Intra-abdominal with unknown origin	3 (7.7%)
	Retroperitoneal	1 (2.6%)
	Lower esophagus	1 (2.6%)
	Rectum	1 (2.6%)
Median size (cm)	Stomach	5
	Duodenum	2.5
	Small bowel	3
	Liver	1
	Intra-abdominal with unknown origin	16
	Retroperitoneal	8
	Lower esophagus	18
	Rectum	2
Presentation	GI bleeding	11 (28.2%)
	Abdominal pain	18 (46.2%)
	Incidental (radiology, endoscopy, post-operatively)	5 (12.8%)
	Other	5 (12.8%)
Type of surgery	Elective	27 (69.2%)
	Emergency	4 (10.3%)
	Not operated	8 (20.5%)
Metastasis	Yes	8 (20.5%)
	No	31 (79.5%)
Recurrance	Yes	5 (12.8%)
	No	34 (87.2%)
Mitosis (≤5 vs >5 per 50 HPF)	Stomach	15 vs 5
	Duodenum	2 vs 0
	Small bowel	3 vs 3 (2 missing)
	Rectum	0 vs 1
	Missing	8
Immune histochemical assay	DOG-1	28 (71.8%)
	CD 34	20 (51.3%)
	SMA	6 (15.3%)
	DOG-7	4 (10.2%)
	h-Caldesmin	2 (5.1%)
	Demsin	1 (2.6%)
Type of cells in histopathology	Spindle-cell	25 (64.1%)
	Epitheloid	0 (0%)
	Mixed	8 (20.5%)
	Not conclusive	6 (15.4%)

Baseline and tumor characteristics

Table 2 compares baseline and tumor characteristics between patients who presented with bleeding and other presentations. Neither group had any differences regarding age, gender, tumor location, size, cluster of differentiation (CD) markers, histological types, and shape. Emergency surgery was required more frequently in patients presented with bleeding (P= 0.001).

**Table 2:** Comparison of baseline characteristics between patients who presented with bleeding versus other presentations

Factor	Type of Presentation		P-Value
	Non-bleeding GIST (n=28)	Bleeding GIST (n=11)	
Age (years)	58.14± 12.46	58.18± 16.01	0.993
Gender			0.648
Male	14 (73.7%)	5 (26.3%)	
Female	15 (53.57%)	5 (45.45%)	
Tumor location			0.057
Gastric	17 (60.71%)	6 (54.55%)	
Duodenal	0	2 (18.18%)	
Jejunum	2 (7.14%)	2 (18.18%)	
Ilium	3 (10.71%)	1 (9.09%)	
Others	6 (21.43%)	0	
Metastasis	4 (14.29%)	3 (27.27%)	0.379
Tumor size (cm) (largest diameter)	3 (1.5- 9.5)	5.3 (4.4- 7)	0.250
Mitosis (>5/high power field)	9/24 (37.50%)	3/9 (33.33%)	>0.99
CD markers	(n= 28)	(n= 10)	
S100	18 (64.29%)	5 (50%)	0.428
DOG	23 (82.14%)	6 (60%)	0.205
SMA	14 (50%)	5 (50%)	>0.99
Desmin	12 (42.86%)	4 (40%)	>0.99
CD34	17 (60.71%)	6 (60%)	>0.99
CD117	25 (92.59%)	10 (100%)	>0.99
Emergency surgery	0	5/10 (50%)	0.001
Histological type			0.692
Spindle	17/24 (70.83%)	8/10 (80%)	
Mixed	7/24 (29.17%)	2/10 (20%)	
Tumor shape			0.079
Exophytic	10/15 (66.67%)	2/7 (28.57%)	
Endophytic	3/15 (20%)	5/7 (71.43%)	
Mixed	2 (13.33%)	0	

The operation was performed endoscopically (one patient in each group), laparoscopic (13 vs. 7 in no bleeding vs. bleeding), open (7 vs. 1 in no bleeding vs. bleeding), and Whipple was performed in two patients (one in each group). There was no difference in the type of surgical procedure between groups (P = 0.440).

Outcomes

Esophagogastroduodenoscopy (EGD) evidence of bleeding occurred in two patients: one in non-bleeding (3.85%) versus one in bleeding (10%) (P = 0.484). Postoperative hemoglobin was 11.8 ± 1.87 mg/dl in the non-bleeding group versus 9.93 ± 1.85 in the bleeding group (P = 0.024).

**Figure 1:**
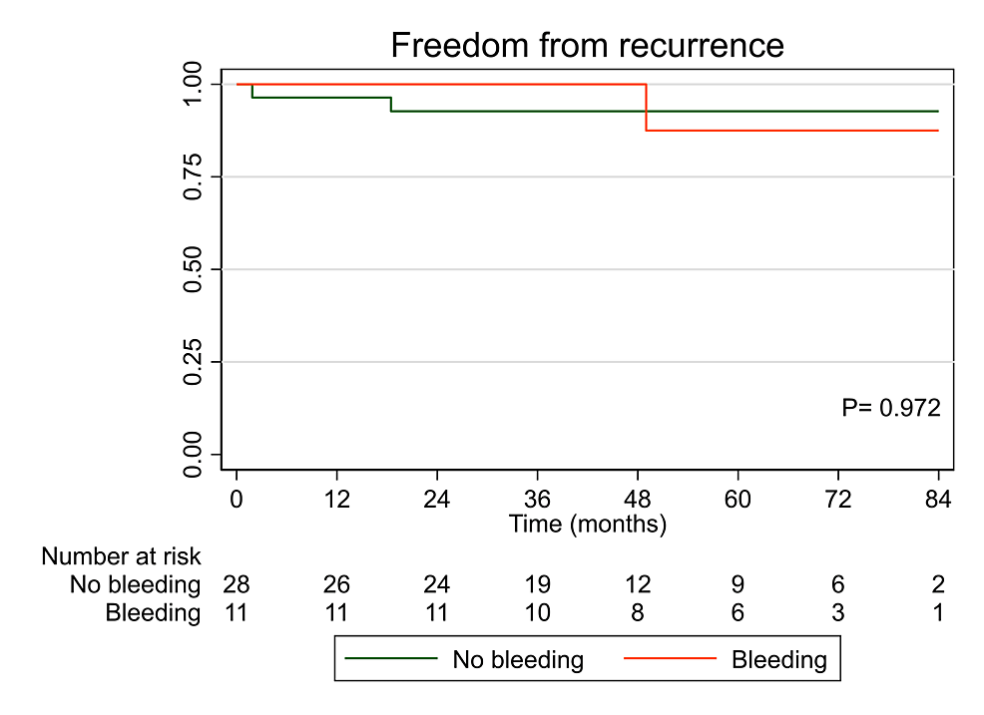
Kaplan-Meier curve for the freedom from recurrence in patients presented with bleeding versus no bleeding

Long-term outcomes:

Figure 1 shows the Kaplan-Meier curve for the freedom from recurrence in patients presented with bleeding versus no bleeding. The median follow-up time was 50 months (range: 36-70 months). Recurrence occurred in five patients (four in non-bleeding presentation and one in bleeding presentation). There was no significant difference in recurrence between both groups (log-rank P = 0.972). Freedom from recurrence at seven years was non-significantly higher in patients with no bleeding (93% in the bleeding group vs. 88% in the non-bleeding group). 

**Figure 2:**
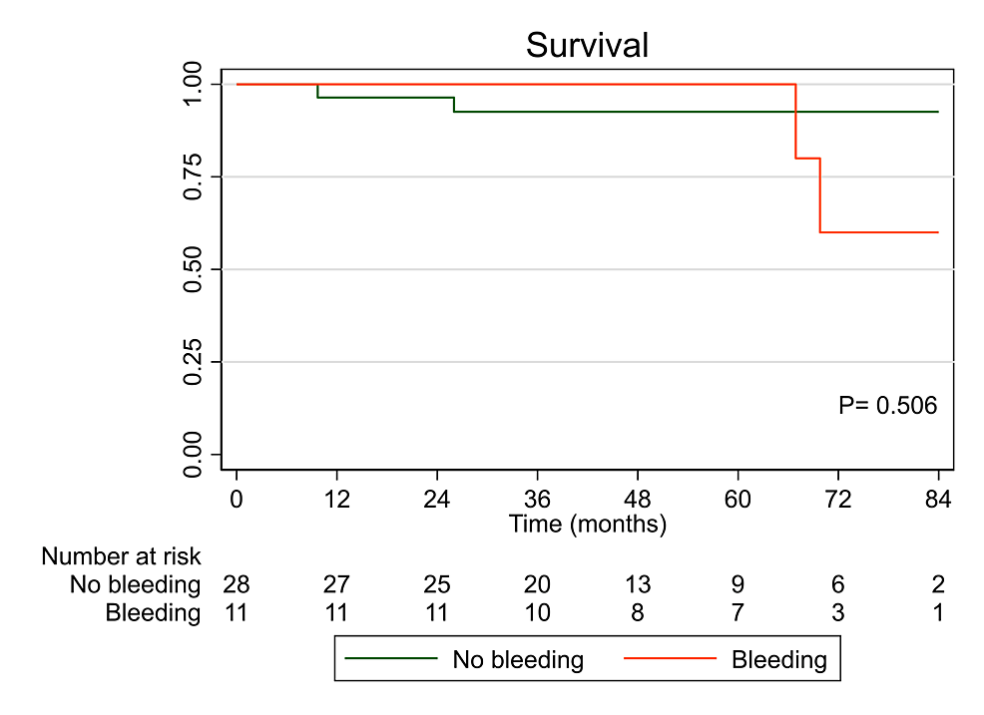
Kaplan-Meier survival curve in patients presented with bleeding vs. no bleeding

Figure 2 shows the Kaplan-Meier survival curve in patients presented with bleeding versus no bleeding. Death at follow-up occurred in four patients, two in each group. There was no difference in survival between both groups (log-rank P = 0.506). Survival at seven years was not significantly lower in patients with bleeding (92% in the non-bleeding group vs. 61% in the bleeding group).

Table 3 analyzes the factors associated with recurrence and mortality. There was no significant association between the baseline characteristics and mortality. Increased tumor size was associated with a higher recurrence rate (HR: 1.29 (95% CI: 1.02-1.62); P = 0.031).

**Table 3:** Univariable Cox regression analysis for factors associated with recurrence and mortality

Risk Factor	Recurrence		Mortality	
	HR (95% CI)	P-Value	HR (95% CI)	P-Value
Age (years)	0.90 (0.78-1.04)	0.161	1.08 (0.98-1.19)	0.116
Female	0.47 (0.04-5.16)	0.534	2.26 (0.23-22.32)	0.485
Tumor location	1.26 (0.63-2.53)	0.512	1.42 (0.75-2.71)	0.283
Metastasis	2.74 (0.25-30.35)	0.412	4.99 (0.69-35.72)	0.109
Tumor size (cm)	1.29 (1.02-1.62)	0.031	0.80 (0.53-1.20)	0.273
CD markers				
S100	-	1.41 (0.13-15.56)	-	0.779
DOG	-	0.20 (0.02-2.25)	-	0.190
SMA	-	0.63 (0.06-7.13)	-	0.710
Desmin	0.67 (0.06-7.35)	0.59 (0.05-6.50)	-	0.664
CD34	1.50 (0.14-16.64)	2.06 (0.17-24.42)	-	0.567
CD117	-	-	-	-
Emergency surgery	4.65 (0.29-74.89)	0.279	-	-
Histological type	2.47 (0.74-8.20)	0.140	-	-
Tumor shape	-	-	1.06 (0.08-13.57)	0.963
Bleeding	1.03 (0.09-11.56)	0.980	1.93 (0.27-13.87)	0.513

## Discussion

Several parameters are used to predict the outcome and prognosis of GIST, such as KIT gene and PDGFRA polypeptide mutations, size and location of the mass, and mitotic rate. However, these parameters are not consistently efficient predictors, which highlights the need to study more prognostic factors [6-9]. In our study, tumor size was significantly associated with a higher recurrence rate, consistent with the literature. On the other hand, the location of the tumor was not significantly associated with a higher recurrence rate. Although not statistically significant, bleeding GIST had higher recurrence and mortality rates. This finding is not consistent with the results of Liu et al. [10]. This may be correlated with our smaller sample size and the fact that patients with bleeding GIST presumably undergo emergency surgery more frequently, which might influence the ability to achieve an R0 resection, leading to a relatively higher recurrence rate. The incidence of bleeding GIST was consistent with the literature. Bleeding was more frequent in larger tumor sizes. In terms of metastasis incidence, there was almost no difference between bleeding and non-bleeding groups, despite the belief that bleeding results from the rupture of the tumor, subsequently leading to the invasion of nearby structures and eventually to metastasis. We concluded that bleeding GIST was higher in gastric GIST, which might be explained by the relatively richer blood supply in the stomach. In contrast to our conclusion, multiple studies suggest that the smaller lumen of the intestine is associated with more mucosal injury, tumor rupture, and bleeding [11,12]. In the literature, there was no gender risk for bleeding, which is consistent with the findings of our study. In a study done by Holmebakk et al., tumor rupture was categorized as major and minor. Major rupture was defined as mass rupture or bowel perforation, while minor rupture was defined as tumor penetration into the peritoneum. In their studies, only major rupture was associated with a higher recurrence rate. [13, 14]. This classification can be taken into consideration in future studies to evaluate rupture and bleeding in GIST as risk factors for both recurrence and mortality.

Even if R0 resection was achieved for GIST, patients tend to have a high recurrence rate (40-50%). However, this rate improved with the introduction of imatinib [12,13,15]. More efforts are needed to study additional parameters that can be involved in stratifying the risk of GIST, as this will help in tailoring the optimal medical and surgical plan for each patient.

Limitation

Our study has its own limitations that should be avoided by future researchers. Firstly, This study only included patients from a single center belonging to the Saudi Ministry of Defense. Therefore, the results yielded in this paper should not be generalized. Secondly, despite the limited prevalence of GIST, a larger sample size than ours (n = 39) would have yielded more accurate results.

## Conclusions

In our study, bleeding GIST was not a significant predictor of recurrence rate or higher mortality. This can help in the debate of whether bleeding GIST should be added as an independent factor in risk stratification. Despite saying that, further studies are needed to study more variables that can add more accuracy to the pre-existing risk stratification systems.
